# Electric Field-Enhanced Cadmium Accumulation and Photosynthesis in a Woody Ornamental Hyperaccumulator—*Lonicera japonica* Thunb.

**DOI:** 10.3390/plants11081040

**Published:** 2022-04-11

**Authors:** Zhouli Liu, Qinglin Chen, Maosen Lin, Mengdi Chen, Cong Zhao, Qingxuan Lu, Xiangyu Meng

**Affiliations:** 1 Liaoning Key Laboratory of Urban Integrated Pest Management and Ecological Security, College of Life Science and Engineering, Shenyang University, Shenyang 110044, China; zlliu@syu.edu.cn (Z.L.); lqx0812@syu.edu.cn (Q.L.); mengxy559@syu.edu.cn (X.M.); 2 Forestry Development Service Center of Liaoning, Shenyang 110036, China; faximcql@163.com; 3 College of Water Conservancy, Shenyang Agricultural University, Shenyang 110161, China; 4 Academy of Forest and Grassland Inventory and Planning of National Forestry and Grassland Administration, Beijing 100714, China; bumblebear17@163.com; 5 Forestry Development Service Center of Fushun, Fushun 113006, China; lovezhanyue1314@foxmail.com

**Keywords:** electric fields, cadmium, *Lonicera japonica* Thunb., hyperaccumulator, phytoremediation

## Abstract

The multi-system of electro-phytotechnology using a woody ornamental cadmium (Cd) hyperaccumulator (*Lonicera japonica* Thunb.) is a new departure for environmental remediation. The effects of four electric field conditions on Cd accumulation, growth, and photosynthesis of *L. japonica* under four Cd treatments were investigated. Under 25 and 50 mg L^−1^ Cd treatments, Cd accumulation in *L. japonica* was enhanced significantly compared to the control and reached 1110.79 mg kg^−1^ in root and 428.67 mg kg^−1^ in shoots influenced by the electric field, especially at 2 V cm^−1^, and with higher bioaccumulation coefficient (BC), translocation factor (TF), removal efficiency (RE), and the maximum Cd uptake, indicating that 2 V cm^−1^ voltage may be the most suitable electric field for consolidating Cd-hyperaccumulator ability. It is accompanied by increased root and shoots biomass and photosynthetic parameters through the electric field effect. These results show that a suitable electric field may improve the growth, hyperaccumulation, and photosynthetic ability of *L.* *japonica.* Meanwhile, low Cd supply (5 mg L^−1^) and medium voltage (2 V cm^−1^) improved plant growth and photosynthetic capacity, conducive to the practical application to a plant facing low concentration Cd contamination in the real environment.

## 1. Introduction

With the rapid development of industrialization and urbanization in the past few decades, a growing number of heavy metals are deposited in soil, mainly derived from mining activities, vehicle emissions, and industrial dust, have caused severe harm to human health and the environment [1,2,3,4,5]. Among those heavy metals, cadmium (Cd), one of the most toxic pollutants, has become a global concern due to its high persistency, strong water-solubility, and potential carcinogenicity [6,7,8,9,10]. Soil Cd exceeding the environmental standard not only poses great harm to plants, including leaf chlorosis, growth inhibition, stomatal closure, and photosynthesis inhibition but threatens human health through the food chains [11,12,13,14,15,16,17]. It is consequently urgent to develop a more efficient technique for removing Cd from contaminated soils [18].

Phytoremediation—hyperaccumulator or accumulator absorption of toxic heavy metals from soils to plant organs—has become a promising technique that is inexpensive, easily applied, and eco-friendly [19,20,21]. The hyperaccumulators have been considered to extract and accumulate Cd above 0.01% dry tissue (100 μg g^−1^) [22,23,24]. However, phytoremediation also shows such typical limitations in practice as deep treatment zone, long-time consumption, and low bioavailability of soil pollutants [25,26]. Recently, the combination of phytoremediation and electrokinetic remediation has increasingly been used to overcome the limitations of phytoremediation and enhance remediation efficiency [27,28,29,30,31,32]. Some studies have reported that the application of electric fields could improve the bioavailability of pollutants in soils and heavy metal accumulation in plants [28,33,34,35], and other studies investigated electric field-assisted enhancements in seed germination, plant growth, and self-organization ability under different environmental stress [36,37,38,39,40]. Nevertheless, these studies were mainly focused on crops, herbs, and aquatic plants, including lettuce, maize, tomato, ryegrass, and Canadian waterweed [41,42,43,44,45,46,47,48]. Early researchers indicated Cd-induced changes in photosynthesis, such as net photosynthesis (Pn), stomatal conductance (Gs), and transpiration rate (Tr) [49,50,51,52,53,54,55,56]. However, little information is available on the electric field-assisted effects on the characteristics of cadmium accumulation and photosynthesis in woody ornamental hyperaccumulators.

Nowadays, more and more ornamental plants are widely used for gardening and greening across the human living environment [57,58,59]. Ornamental plants not only clean up the soil contaminated by heavy metals but also contribute to the beautification and decoration of the living environment [60,61]. *Lonicera japonica* Thunb.—a popular woody ornamental plant—has become established in temperate and tropical regions worldwide in the past 150 years [62]. The plant has the characteristics of easy cultivation, high biomass, wide geographic distribution, and strong resistance to environmental stress [63]. *L. japonica* was chosen in the study based on our previous findings, which showed that it is a new-found woody Cd-hyperaccumulator [53,64,65]. Therefore, in the present study, we selected *L. japonica* as a model plant to show the effect of different electric fields on Cd accumulation and transport, and investigate the responses of plant growth and photosynthesis under different Cd concentrations. The specific objectives are to confirm the phytoremediation potential of a woody ornamental Cd-hyperaccumulator assisted by electric fields and develop a practical multi-system of electro-phytotechnology used to prevent contaminated soils.

## 2. Materials and Methods

### 2.1. Plant Cultivation and Treatments

Seedlings of *L. japonica* were collected from the non-contaminated experimental field of Shenyang Agricultural University and propagated in sterilized sand with a nutrient medium. The nutrient medium was Hoagland solution modified by the following composition (mmol L^−1^): Ca(NO_3_)_2_ × 4 H_2_O 5.00, MgSO_4_ × 7 H_2_O 2.00, KNO_3_ 5.00, KH_2_PO_4_ 1.00, H_3_BO_3_ 0.05, ZnSO_4_ × 7 H_2_O 0.80 × 10^−3^, MnCl_2_ × 4 H_2_O 9.00 × 10^−3^, CuSO_4_ × 5 H_2_O 0.30 × 10^−3^, (NH_4_)_6_Mo_7_O_24_ × 4 H_2_O 0.02 × 10^−3^, Fe-EDTA 0.10 [64,66]. The pH was adjusted daily to 5.8 ± 0.1 with HCl or NaOH. The plants were grown in a greenhouse of Shenyang Agricultural University at 23 ± 2 °C (800–1000 μmol m^−2^ s^−1^ PPFD, 16/8 h light/dark, 70–80% relative humidity).

**Figure 1:**
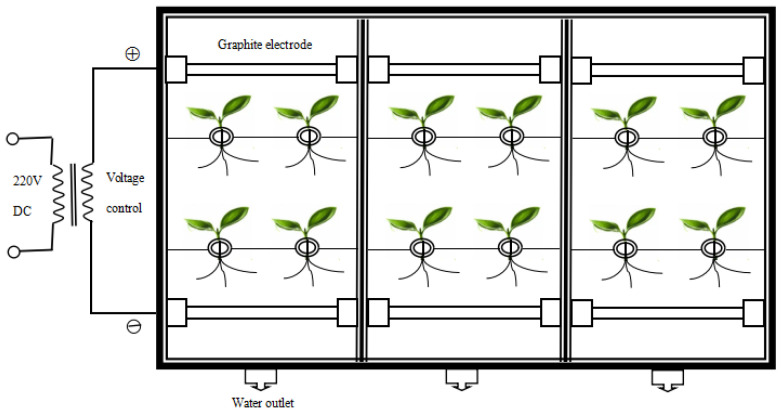
The electrical setting of the experiment.

After 8 weeks of cultivation, *L. japonica* were transferred into adumbral containers (45 × 22 × 15 cm^3^) with 6 L Hoagland nutrient medium, 4 plants for each. The nutrient medium was renewed once every 3 days. Subsequently, Cd^2+^ (CdCl_2_ × 2.5 H_2_O, Kermel Chemical Reagent Co., Ltd., Tianjin, China, >99%) was added into the nutrient medium to get: 0, 5, 25 and 50 (mg L^−1^), respectively. Additionally, a homogeneous electric field with a pair of graphite electrodes (10.0 cm length, R = 3 mm) connected to a DC power supply (220 V, 50 Hz) was applied for 6 h per day, and the electrical setting of the experiment is shown in Figure 1. The voltage gradients of 0, 1, 2, and 3 (V cm^−1^) are shown in Table 1. The experiment was repeated 3 times, and the plants were harvested 1 week later for analysis.

### 2.2. Measurements of Photosynthetic Parameters

Photosynthetic parameters were measured in fully expanded leaves under the electric field using a portable photosynthesis system (LI-6400, Li-Cor Inc. Lincoln, NE, USA). The photosynthetic parameters contained net photosynthetic rate (Pn, μmol m^−2^ s^−1^), stomatal conductance (Gs, mol m^−2^ s^−1^), transpiration rate (Tr, mmol m^−2^ s^−1^), and intercellular CO_2_ concentration (Ci, µL L^−1^). Light level, CO_2_ concentration, and leaf temperature inside the leaf chamber were kept constant at 1000 μmol m^−2^ s^−1^ PPFD, 25 ± 0.3 °C, and 380 ± 5 μmol CO_2_ mol^−1^, respectively. Eight leaves per treatment were used for the determination.

### 2.3. Assays of Plant Biomass and Cd Content

After harvesting, *L. japonica* were washed with tap water, and the plant roots were immersed in 20 mM Na_2_-EDTA for 15 min and then rinsed with tap and de-ionized water to remove Cd adhering to the root surface. The plants were separated into shoots and roots. These portions were then dried at 105 °C for 20 min, then at 70 °C until a constant weight was reached. Afterward, root and shoots biomass dry weight was obtained.

Dried plant materials were ground to a fine powder. The powders were digested with a concentrated acid mixture of HNO_3/_HClO_4_ (3:1, *v*/*v*). The Cd concentration in plant tissues was determined using an atomic absorption spectrophotometer (AAS 3110 Perkin-Elmer, Waltham, MA, USA).

### 2.4. Data Analysis

The bioaccumulation coefficient (BC) indicated the ability of plants to accumulate cadmium in the medium. It was shown as:

(1) $$\mathrm{BC}=\frac{\mathrm{the}\;\mathrm{cadmium}\;\mathrm{concentration}\;\mathrm{in}\;\mathrm{the}\;\mathrm{plant}}{\mathrm{the}\;\mathrm{cadmium}\;\mathrm{concentration}\;\mathrm{in}\;\mathrm{the}\;\mathrm{solution}}$$

The translocation factor (TF) reflected the different abilities of plants to translocate cadmium from a different portion of plants. It was described as:

(2) $$\mathrm{TF}=\frac{\mathrm{the}\;\mathrm{cadmium}\;\mathrm{concentration}\;\mathrm{in}\;\mathrm{shoots}}{\mathrm{the}\;\mathrm{cadmium}\;\mathrm{concentration}\;\mathrm{in}\;\mathrm{roots}}$$

To assess the effects of cadmium on phytoextraction by plants, the removal efficiency (RE) was described as [19,67].

(3) $$\mathrm{RE}=\frac{\left({\mathrm{Metal}}_{\mathrm{shoot}}\times {\mathrm{Mass}}_{\mathrm{shoot}}\right)}{\left({\mathrm{Metal}}_{\mathrm{medium}}\times {\mathrm{Mass}}_{\mathrm{medium}}\right)}$$

where Metal_shoot_ is the contents (mg kg^−1^) of cadmium in the harvested shoots of plants; Metal_medium_ is the initial cadmium content (mg kg^−1^) of the medium; Mass_shoot_ and Mass_medium_ are the masses (g) of the shoots and medium of the harvested plants, respectively.

The cadmium uptake was measured as [65].

(4) $$\mathrm{Uptake}\left(\mathrm{\mu g}\;{\mathrm{plant}}^{-1}d^{-1}\right)=\frac{M_{2}W_{2}-M_{1}W_{1}}{T_{2}-T_{1}}$$

where M_1_ and M_2_ are the cadmium concentrations in the plant tissue, and W_1_ and W_2_ are the plant biomass at time T_1_ (initial sampling) and T_2_ (final sampling).

### 2.5. Statistical Analyses

All experimental measurements were set for three replicates. Average values and standard deviations (SD) were calculated by Microsoft Office Excel 2016 for all the data in the present study. The experimental data were presented as the means ± SD. The statistical analysis of variance was carried out with the SPSS 22.0 software tool. The significant difference was performed between treatments at *p* < 0.05. Multiple comparison was also determined using the least significant difference (LSD) test.

## 3. Results and Discussion

### 3.1. The Effect of Electric Field on Cd Accumulation in Plants

**Figure 2:**
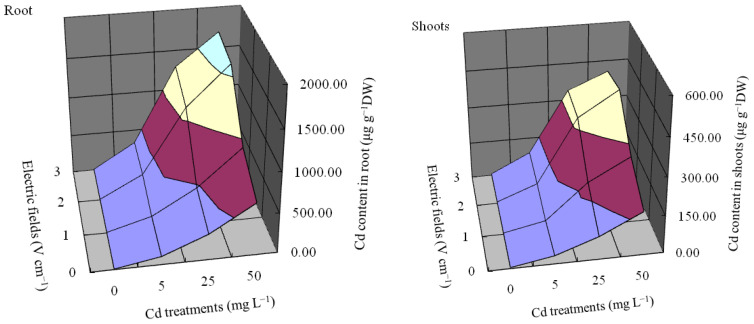
The relationship of different treatments and Cd contents in roots and shoots of *L.* *japonica*. Values represent the mean.

Under different treatments, Cd accumulation in roots and shoots of *L.* *japonica* was shown in Figure 2. Under T_1_–T_4_ treatments (under Cd stress without electric field), Cd concentration in roots had a slightly increasing trend which ranged from 103.72 to 657.58 mg kg^−1^. Under T_5_–T_16_ treatments, the electric field significantly enhanced the concentrations of Cd in roots compared with the control, especially exposed to high concentrations (25 and 50 mg L^−1^) Cd. The Cd concentrations in roots were enhanced significantly by 2 and 3 V cm^−1^ voltages, which reached 1110.79 and 1608.24 mg kg^−1^ (T_11_ and T_12_), 1291.95 and 1692.37 mg kg^−1^ (T_15_ and T_16_), respectively. Despite that, Cd concentrations in shoots were enhanced significantly in the electric field. The different voltages promoted Cd concentrations in shoots above 180 mg kg^−1^ under 25 and 50 mg L^−1^ Cd stress. The maximum Cd concentrations in shoots increased by 2 V cm^−1^ voltage (exposed to 50 mg L^−1^ Cd) were added to 428.67 mg kg^−1^, 2.44 times of T_4_ treatment (V0-Cd50). Other studies also reported that heavy metal (Cd, Cu, Zn, and Pb) concentrations in plants increased with the application of an electric field [29,68]. The beneficial effect of the electric field may be associated with the changes in cell membrane properties and metal ions polarity inside plants [42,45]. Our present study is in agreement with the observation of [35,46], which showed that electric fields could enhance the rate of membrane polarization and cell metabolism, which accelerate heavy metal transport by activating ion channels such as Ca^2+^ into cytosol and enzyme cascades.

### 3.2. The Effect of Electric Field on Hyperaccumulation Characteristics

The measured results of the bioaccumulation coefficient (BC), translocation factor (TF), removal efficiency (RE), and heavy metal uptake are shown in Table 2. The values of BC, TF, RE, and heavy metal uptake refer to the plant’s characteristic to absorb metal elements from the soil, then transfer, mobilize and store these elements in plant tissues [38,47,69,70,71,72]. Therefore, these results, including BC, TF, RE, and Cd uptake, are very important to understanding the action of ion exchange in the soil environment and hyperaccumulation characteristics in the tissues of *L*. *japonica*. The electric fields enhanced root BC significantly and shoots BC of *L*. *japonica* exposed to different concentrations of Cd compared with T_1_–T_4_ treatments (under Cd stress without electric fields). Under different concentrations of Cd stress, root BC and shoots BC were promoted significantly by 2 V cm^−1^ voltage and 3 V cm^−1^ voltage, which reached above 32.16 (T_12_) and 8.11 (T_16_). When exposed to higher concentrations of Cd, the TF of the plants was enhanced significantly by 1 V cm^−1^ voltage and 2 V cm^−1^ voltage. Exposed to 5 mg L^−1^ Cd stress, the RE of the plants was increased from 14.02 under no voltage (T_2_) treatment to 36.78 under 3 V cm^−1^ voltage treatment (T_14_). With the increase of Cd concentration in the medium, the maximum RE in the plants was 4.00 and 2.61 times of T_3_ (V0-Cd25) and T_4_ treatment (V0-Cd50). Compared with the treatments (under Cd stress without electric field), different voltages enhanced the uptake of Cd in the plants exposed to different concentrations of Cd. The maximum Cd uptake of the plants reached 53.02 μg plant^−1^ day^−1^ promoted by 2 V cm^−1^ voltage (T_11_).

### 3.3. The Effect of Electric Field on Root and Shoots Dry Weight

**Figure 3:**
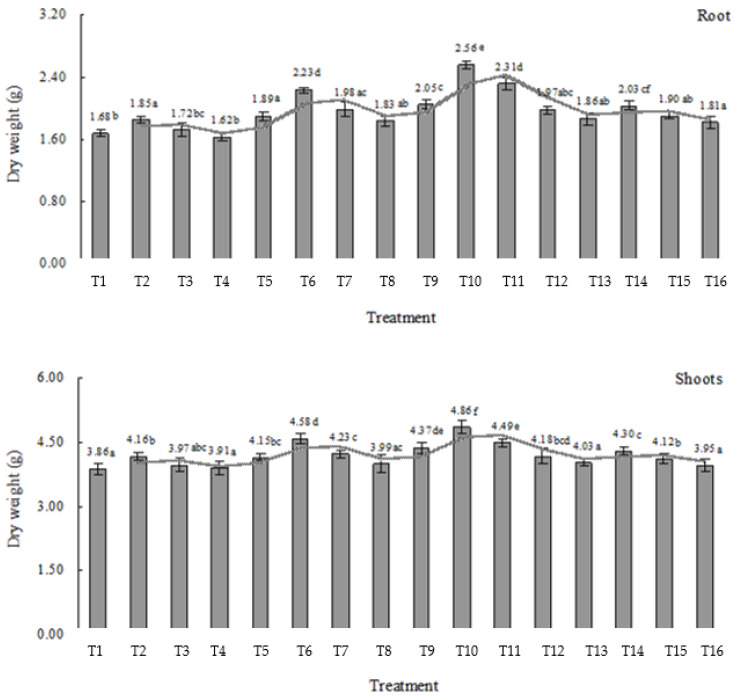
The effect of electric field on root and shoots biomass dry weight (g) of *L.* *japonica*. Values represent mean ± SD. Different letters indicate significant differences at the 5% level according to the LSD test.

The root and shoots biomass of the plant are considered highly sensitive indicators in their response to heavy metal and other environmental stress [37,73,74,75]. The growth responses of *L.* *japonica* in terms of root and shoots biomass dry weight under different treatments are shown in Figure 3. Under T_1_–T_4_ treatments (under Cd stress without electric field), the dry weight of root biomass increased exposed to 5 and 25 mg L^−1^ Cd, and decreased slightly exposed to 50 mg L^−1^ Cd, indicating that the plants had a good tolerance to Cd stress. Under T5, T9, and T13 treatments (under electric field without Cd stress), the dry weight of root biomass significantly increased, especially under 2 V cm^−1^ voltage (T_9_), 22.02% higher than the control. Under T_6__–8_, T_10__–12,_ and T_14__–16_ treatments (under electric field and Cd stress), different voltages enhanced the dry weight of root biomass exposed to different concentrations of Cd compared to the control. Furthermore, under 2 V cm^−1^ voltage, the dry weight of root biomass exposed to 5 (T_10_) and 25 mg L^−1^ Cd (T_11_) were enhanced significantly by 52.38% and 37.50% higher than the control. A similar phenomenon [43] also shows that the application of a pulsed electric field increased the root dry weight of maize under drought stress, and the enhancement could result from the improved respiration metabolism under the influence of the pulsed electric field to affect the synthesis and substance transformation. When the plants were exposed to 50 mg L^−1^ Cd stress, different voltages promoted the dry weight of root biomass compared with T_4_ treatment (V0-Cd50).

By comparison, under T_1_–T_4_ treatments (under Cd stress without electric field), the dry weight of shoots biomass increased when exposed to different concentrations of Cd. The earlier study [42] showed that the stronger electric field had a limited influence on the growth of hydroponically cultivated tomatoes. However, our results divulged that under T5, T9, and T13 treatments (under electric field without Cd stress), the dry weight of shoots biomass increased significantly, especially under 2 V cm^−1^ voltage (T_9_), 13.21% higher than the control. It agrees with the study [30], which reported that *Brassica rapa* L. showed fast growth and biomass production with a low or moderate voltage gradient. Under T_6__–8_, T_10__–12,_ and T_14__–16_ treatments (under electric field and Cd stress), there is a similarly increased trend regarding the dry weight of root biomass. Furthermore, under 2 V cm^−1^ voltage, the dry weight of shoots biomass exposed to 5 mg L^−1^ Cd (T_10_) was enhanced significantly by 25.91% higher than the control. When the plants were exposed to 50 mg L^−1^ Cd stress, the dry weight of shoots biomass was enhanced by different voltages compared with T_4_ treatment (V0-Cd50), which showed the electric field could promote the tolerance responses of the plants to high concentrations of Cd.

Above all, applying a medium-strength electric field enhanced the root and shoots growth of *L.* *japonica.* In the present study, under 2 V cm^−1^ voltage, the dry weight of root and shoots biomass was significantly enhanced to 5 mg L^−1^ Cd compared with the control. Our present results agree with previous reports proving that the electric field stimulated plant biomass production and growth by regulating the transport and distribution of plant growth hormones [27,28,33,42,46].

### 3.4. The Effect of Electric Field on Photosynthetic Parameters

**Figure 4:**
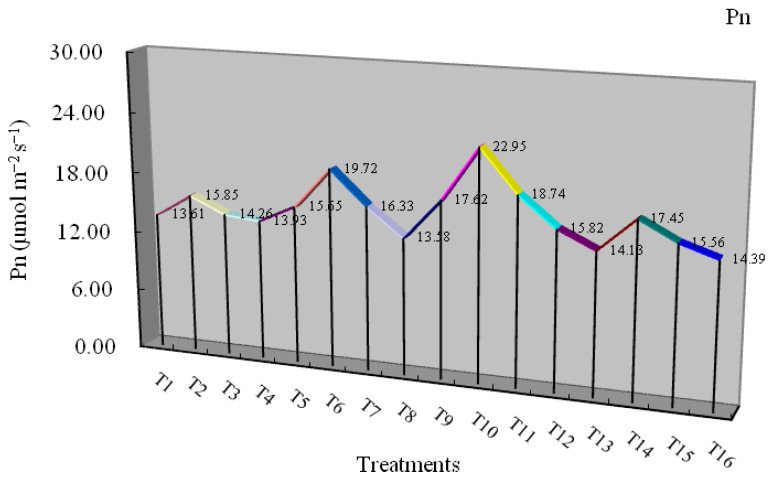

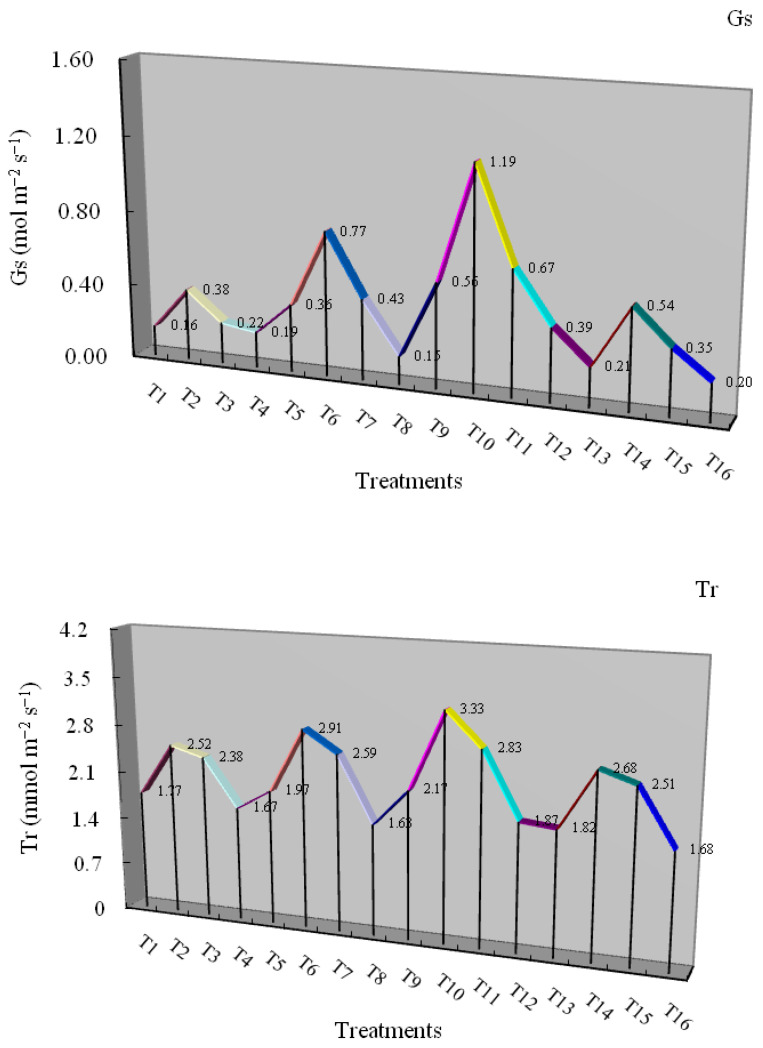

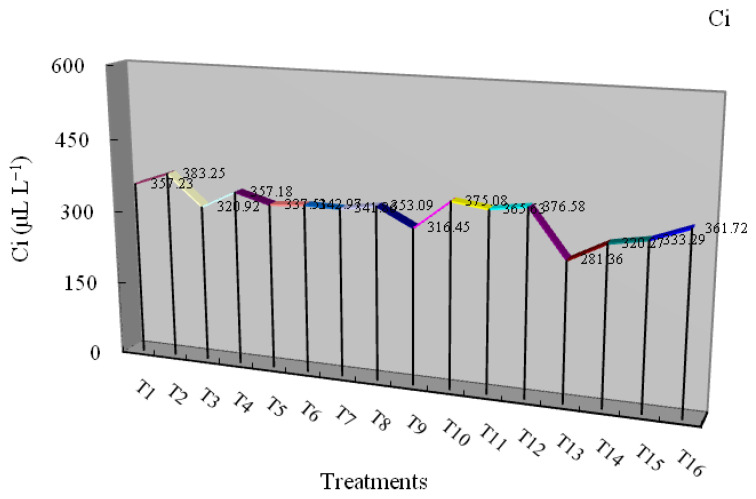
The effect of electric field on net photosynthetic rate (Pn), stomatal conductance (Gs), transpiration rate (Tr), and intercellular CO_2_ concentration (Ci). Values represent the mean.

Photosynthesis, one of the most important biological processes for plant growth and food production, is especially sensitive to Cd stress [76,77,78,79]. As shown in Figure 4, the net photosynthetic rate (Pn), stomatal conductance (Gs), transpiration rate (Tr), and intercellular CO_2_ concentration (Ci) in the leaves of *L.* *japonica* under different treatments were evaluated. The significant stimulating effect of Pn exposed to low concentrations of Cd resulted in the improvement of gas exchange and transpiration in terms of the increase in Gs, Tr, and Ci, indicating the positive effect of low Cd concentrations on the contents of Rubisco [22]. A similar phenomenon was also found in maize plants [49]. The Pn, Gs, and Tr of the plants exposed to Cd stress concentrations had a similar response visualized as an inverted U-shaped curve under different voltages. Under different concentrations of Cd stress, the maximum Pn, Gs, and Tr of the plants reached 22.95 μmol m^−2^ s^−1^, 1.19 mol m^−2^ s^−1,^ and 3.33 mmol m^−2^ s^−1^, and all were enhanced significantly by 2 V cm^−1^ voltage (T_10_, V2-Cd5). It is coincident with root and shoots biomass dry weight, indicating the combination of the low Cd supply (5 mg L^−1^) and medium voltage (2 V cm^−1^) was beneficial to elevate the photosynthetic capacity and growth of the plants. A similar phenomenon is also described as the hormesis effect [53]. When the plants were exposed to higher concentrations (25 mg L^−1^), different voltages significantly enhanced the Pn, Gs, Tr, and Ci of the plants. However, with the increase of Cd concentration in the medium, the effects were not obvious compared with T_3_ treatment (V0-Cd25; *p* < 0.05). Some studies reported that Cd negatively affected photosynthesis [80,81,82], which may be attributed to the inhibition of chlorophyll biosynthesis, pigment-protein complexes, thylakoids, or reduction in growth [80,83,84]. However, in our present study, increased or unimpacted photosynthesis showed that *L.* *japonica* did not suffer metal toxicity and produced metabolites for absorption, defense, growth, and development, which may be related to good tolerance and hyperaccumulation characteristics of the plant to stress [85]. Under T_13_-T_16_ treatments, the increased Ci with the increase of Cd concentration in the medium may result from inhibiting chloroplast metabolism via hampering light and dark reactions of photosynthesis or Calvin cycle enzymes and the photosynthetic electron transport chain [86].

## 4. Conclusions

In the present study, the electric field significantly enhanced the concentrations of Cd in roots and shoots compared with the control, especially exposed to high concentrations (25 and 50 mg L^−1^) Cd. The Cd concentrations in roots were enhanced significantly by 2 and 3 V cm^−1^ voltages, which reached above 1110.79 mg kg^−1^ (T_11_ and T_12_) and nearly 1700.00 mg kg^−1^ (T_15_ and T_16_), respectively. By comparison, the different voltages promoted Cd concentrations in shoots above 180 mg kg^−1^ under 25 and 50 mg L^−1^ Cd stress. The maximum Cd concentrations in shoots increased by 2 V cm^−1^ voltage (exposed to 50 mg L^−1^ Cd) were added to 428.67 mg kg^−1^, which was 2.44 times higher than T_4_ treatment (V0-Cd50). Root BC, shoots BC, TF, and the maximum Cd uptake of the plant were also promoted significantly by 2 V cm^−1^ voltage. The above results showed that 2 V cm^−1^ voltage might be the most suitable electric field for improving the hyperaccumulation ability of the plant.

At the same time, under 2 V cm^−1^ voltage, the root and shoots biomass dry weight of *L.* *japonica* exposed to 5 mg L^−1^ Cd increased significantly compared to the control. The maximum Pn, Gs, and Tr of the plants exposed to different concentrations of Cd were also enhanced significantly by 2 V cm^−1^ voltage (T_10_, V2-Cd5), which indicated that the synergistic benefits of low Cd supply (5 mg L^−1^) and medium voltage (2 V cm^−1^) contributed to the elevated plant growth and photosynthetic capacity. This characteristic is conducive to the practical application of the plant facing low concentration Cd-contamination in the real environment. Under T_1_-T_4_ treatments (under Cd stress without electric field), the dry weight of root biomass decreased slightly exposed to 50 mg L^−1^ Cd, indicating the good tolerance of the plant to Cd. However, under T_6__–8_, T_10__–12,_ and T_14__–16_ treatments (under electric field and Cd stress), different voltages promoted the dry weight of root biomass compared with T_4_ treatment (V0-Cd50), indicating the electric field could promote the tolerant ability of the plant to higher concentration Cd.

Moreover, different voltages significantly enhanced the Pn, Gs, Tr, and Ci of the plants exposed to 25 mg L^−1^ Cd. With the increase of Cd concentration in the medium, the effects were not obvious compared with T_3_ treatment (V0-Cd25). Increased or unimpacted photosynthesis showed that L. japonica did not suffer metal toxicity and produced metabolites for absorption, defense, growth, and development, along with the improved growth tolerance and hyperaccumulation ability of the plant in the electric field under external Cd stress.

According to the results above, on the one hand, it is feasible to develop a multi-system of electro-phytotechnology using a woody Cd-hyperaccumulator (*L. japonica*) to remediate Cd contamination. On the other hand, *L. japonica*, as a popular ornamental, had dual merits of phytoremediation and decoration, which will bring social and environmental benefits. The present study would provide a reference for promoting large-scale soil remediation by electric field-assisted phytoremediation, and future researchers need to consider the economic feasibility.

## Figures and Tables

**Table 1:** Various experimental treatments.

Treatment	Test Number	Voltage Gradient (V cm^−^^1^)	Cd Concentration in the Medium (mg L^−1^)
T1	V0-Cd0	0	0
T2	V0-Cd5	0	5
T3	V0-Cd25	0	25
T4	V0-Cd50	0	50
T5	V1-Cd0	1	0
T6	V1-Cd5	1	5
T7	V1-Cd25	1	25
T8	V1-Cd50	1	50
T9	V2-Cd0	2	0
T10	V2-Cd5	2	5
T11	V2-Cd25	2	25
T12	V2-Cd50	2	50
T13	V3-Cd0	3	0
T14	V3-Cd5	3	5
T15	V3-Cd25	3	25
T16	V3-Cd50	3	50

**Table 2:** The effect of electric field on Cd hyperaccumulation characteristics of *L.* *japonica*.

Treatment	Test Number	Root BC	Shoots BC	TF	RE	Uptake (μg plant^−1^ day^−1^)
T1	V0-Cd0	—	—	—	—	—
T2	V0-Cd5	20.74 ^a^	6.74 ^ab^	0.33 ^a^	14.02 ^ab^	5.43 ^abc^ ± 0.04
T3	V0-Cd25	13.04 ^b^	3.72 ^c^	0.29 ^bc^	7.39 ^c^	10.68 ^d^ ± 0.08
T4	V0-Cd50	13.15 ^b^	3.51 ^d^	0.27 ^d^	6.87 ^d^	14.68 ^ef^ ± 0.06
T5	V1-Cd0	—	—	—	—	—
T6	V1-Cd5	31.85 ^cd^	7.94 ^cde^	0.25 ^b^	18.18 ^ef^	8.95 ^de^ ± 0.05
T7	V1-Cd25	18.92 ^e^	7.27 ^ab^	0.38 ^a^	15.37 ^ab^	20.86 ^g^ ± 0.12
T8	V1-Cd50	18.61 ^e^	5.40 ^e^	0.29 ^bc^	10.77 ^g^	19.18 ^gh^ ± 0.23
T9	V2-Cd0	—	—	—	—	—
T10	V2-Cd5	57.93 ^ef^	13.73 ^cdef^	0.24 ^b^	33.36 ^h^	18.46 ^ghi^ ± 0.11
T11	V2-Cd25	44.43 ^g^	13.17 ^de^	0.30 ^a^	29.56 ^i^	53.02 ^j^ ± 0.38
T12	V2-Cd50	32.16 ^cd^	8.57 ^abc^	0.27 ^cd^	17.92 ^efgh^	16.36 ^abcd^ ± 0.15
T13	V3-Cd0	—	—	—	—	—
T14	V3-Cd5	65.81 ^h^	17.10 ^g^	0.26 ^b^	36.78 ^jk^	17.80 ^fg^ ± 0.09
T15	V3-Cd25	51.68 ^efg^	13.56 ^def^	0.26 ^b^	27.92 ^hi^	50.27 ^ijk^ ± 0.40
T16	V3-Cd50	33.85 ^cd^	8.11 ^ab^	0.24 ^bcd^	16.02 ^abc^	14.55 ^cdef^ ± 0.07

Data are means ± SD. BC: the bioaccumulation coefficient; TF: the translocation factor; RE: the removal efficiency. Different letters indicate significant differences at the 5% level according to the LSD test. “—” Unavailable under the tested concentrations.

## Data Availability

The data presented in this study are available on request from the corresponding author. The data are not publicly available due to the restriction policy of the co-authors’ affiliations.
